# Performance of artificial intelligence for the detection of pathological myopia from colour fundus images: a systematic review and meta-analysis

**DOI:** 10.1038/s41433-023-02680-z

**Published:** 2023-08-07

**Authors:** Jai Prashar, Nicole Tay

**Affiliations:** 1 University College London, London, UK; 2 Moorfields Eye Hospital NHS Foundation Trust, London, UK

**Keywords:** Physical examination, Epidemiology

## Abstract

**Background:**

Pathological myopia (PM) is a major cause of worldwide blindness and represents a serious threat to eye health globally. Artificial intelligence (AI)-based methods are gaining traction in ophthalmology as highly sensitive and specific tools for screening and diagnosis of many eye diseases. However, there is currently a lack of high-quality evidence for their use in the diagnosis of PM.

**Methods:**

A systematic review and meta-analysis of studies evaluating the diagnostic performance of AI-based tools in PM was conducted according to the Preferred Reporting Items for Systematic Reviews and Meta-Analyses (PRISMA) guidance. Five electronic databases were searched, results were assessed against the inclusion criteria and a quality assessment was conducted for included studies. Model sensitivity and specificity were pooled using the DerSimonian and Laird (random-effects) model. Subgroup analysis and meta-regression were performed.

**Results:**

Of 1021 citations identified, 17 studies were included in the systematic review and 11 studies, evaluating 165,787 eyes, were included in the meta-analysis. The area under the summary receiver operator curve (SROC) was 0.9905. The pooled sensitivity was 95.9% [95.5%-96.2%], and the overall pooled specificity was 96.5% [96.3%-96.6%]. The pooled diagnostic odds ratio (DOR) for detection of PM was 841.26 [418.37–1691.61].

**Conclusions:**

This systematic review and meta-analysis provides robust early evidence that AI-based, particularly deep-learning based, diagnostic tools are a highly specific and sensitive modality for the detection of PM. There is potential for such tools to be incorporated into ophthalmic public health screening programmes, particularly in resource-poor areas with a substantial prevalence of high myopia.

## Introduction

Myopia is one of the most common ocular conditions worldwide, with global prevalence predicted to increase from nearly 2.8 billion in the year 2020 to almost 5 billion—~49.8% of the world’s population—by the year 2050 [1]. High myopia, generally defined as a refractive error of −6 dioptres (D) or greater, can predispose individuals to sight-threatening sequelae such as glaucoma, cataract, retinal tears or detachment.

Pathological myopia (PM)—which occurs as a result of structural changes in the posterior segment of the eye due to significant axial elongation [2], is one of the major causes of irreversible visual impairment worldwide [2–5], affecting ~3% of the world population and as many as 50–70% of high myopics to some degree [6]. Reduced visual acuity due to PM can result in a considerable negative impact on quality of life, including social and emotional health and functional ability [7]. The potential economic impact of PM is also profound; a 2015 meta-analysis estimated the global productivity loss caused by myopic macular degeneration to be around US $6 billion worldwide [8].

The prevalence of myopia—the main risk factor for PM development—is extreme in many areas; in one study of 23,616 males in South Korea, 96.5% were myopic [9]. Evidence suggests that treatment failure in the correction of myopia is common and that long-term efficacy (of importance in reducing the risk of PM) is often limited [10]. As a result, a significant number of individuals, particularly in highly myopic populations, are still likely to develop PM, underscoring the need for cost-effective, reliable and scalable screening programmes to identify and monitor patients with PM and follow-up those at high risk of developing sight-threatening complications.

The diagnosis of PM is made qualitatively on fundal examination. Qualitative diagnosis can be subject to inter-observer variability between practitioners, and requires considerable clinical expertise to perform accurately. While optical coherence tomography (OCT) may also be used in the diagnosis of PM, retinal fundus photography remains the most widely accessible form of ophthalmic imaging for screening purposes. Hence, at present, retinal fundus images are likely to be the most useful modality to test the efficacy of new diagnostic aids and tools.

Several ‘classical’ features of PM may be visualised on fundus imaging, including a ‘tesselated’ atrophy of the retinal pigment epithelium, peripapillary atrophy, temporal flattening of the optic disc, lacquer cracks, posterior staphyloma and Fuch’s spot. A common cause of blindness in PM is myopic choroidal neovascularisation (CNV), which carries an extremely poor prognosis if untreated [2, 11].

Artificial intelligence (AI)-based diagnostic tools seek to reduce the need for expert interpretation by learning the features of normal and abnormal examples, with the aim of being able to label images autonomously. AI-aided diagnosis is no longer a novel concept in ophthalmology, and has been the subject of much evaluation for the screening of multiple ocular diseases, such as age-related macular degeneration, glaucoma, diabetic retinopathy, papilloedema and retinopathy of prematurity [12–16]. However, no systematic review or meta-analysis to date has sought to collate and evaluate the efficacy of these methods for the diagnosis of PM.

Therefore, the aim of the present systematic review and meta-analysis is to assess the diagnostic accuracy of artificial intelligence-based methods for the detection of PM using colour fundus images.

## Methods

### Study registration

This study was registered on PROSPERO with registration number CRD42022309830.

### Search strategy and inclusion/exclusion criteria

According to the Preferred Reporting Items for Systematic Review and Meta-Analyses (PRISMA), and using a search strategy designed by JP (Supplementary Table 1), the MEDLINE, EMBASE, CINAHL, Web of Science and IEEEExplore databases were searched. Reference lists of included studies were subsequently hand searched to identify additional studies that met the predefined inclusion criteria.

Studies were included if they reported the effectiveness of machine learning- or artificial intelligence-based detection algorithms in detecting PM; used indices such as area under the receiver-operator curve (AUROC), sensitivity and specificity to report on algorithm performance; evaluated colour fundus images; provided information about the size of the dataset and the reference standard; included a validation set at least 10% of the size of the training set; were in English and were published in a peer-reviewed journal. Reviews and conference abstracts were not included.

### Study selection

Both reviewers independently screened all citations (and subsequently the full texts of included citations) for inclusion in a blinded process. Disagreements were resolved via mutual discussion, and details of these disagreements, along with final decisions on inclusion, are included in Supplementary Table 7.

### Data extraction and quality assessment

A single reviewer (NT) extracted data from the included studies (Table 1, 2). Extracted data were directly checked against study data by a second reviewer (JP). Attempts were made to contact study authors for any missing information. Risk of bias assessment was performed using a novel, multi-step approach combining the Quality Assessment of Diagnostic Accuracy Studies 2 (QUADAS-2 [17]) checklist and the Checklist for Artificial Intelligence in Medical Imaging (CLAIM [18]). Each reviewer performed quality assessment independently using both checklists. Areas of conflict were highlighted and are included in Supplementary Table 7.

**Table 1:** Summary of included studies.

Study ID	Study design	Study country	Total study sample size (patients/images)	Training data makeup	Training data source	Test data makeup	Test data source	Open source data	Reference standard	Image type	Camera	Input resolution	Definition
Cen 2021 [35]	Retrospective	China	129,494/249,620	5945 PM 123,319 non-PM	Various private hospital-based datasets + EyePACS [56]	4768 PM 106,462 non-PM	Various private hospital-based datasets + Messidor-2 [57] + PALM [58] and EyePACS [56]	Some	20 ophthalmologists (including senior retinal specialists)	Colour fundus images	Training: Zeiss FF450 Plus IR, Topcon TRC-50DX. Test: Various	299 × 299	NS
Demir 2021 [25]	Retrospective	Turkey	NS/6426	242 PM 6184 non-PM	ODIR [59]	10-fold cross validation	ODIR [59]	Y	Expert ophthalmologists (unknown number)	Colour fundus images	Various: Canon, Zeiss, and Kowa cameras	125 × 125	NS
Du 2021 [30]	Retrospective	Japan	NS/7020^b^	All myopic NS	Private hospital-based dataset (Japan)	All myopic 981 PM 863 non-PM	Private hospital-based dataset (Japan) + PALM [58] and SEED [60, 61]	Some	3 retinal specialists	Colour fundus images	Topcon TRC50DX and Kowa VC-10i	456 × 456	META-PM [8]
Guo 2021 [28]	Retrospective	USA	NS/250	NS	Kaggle Fundus Image1000 dataset [62]	49 PM 201 non-PM	Kaggle FundusImage1000 dataset [62]	Y	Retinal specialists	Colour fundus images	NS	224 × 224	NS.
Li 2021b [31]	Retrospective	China	NS/64914	8243 PM 38,258 non-PM	Various private hospital-based datasets	389 PM 7787 non-PM	Various private hospital-based datasets	N	17 ophthalmologists	Colour fundus images	Various - NS	512 × 512	META-PM
Li 2022 [33]	Retrospective	China	29230/57148	4862 PM 35,732 non-PM (training and validation)	Various private hospital-based datasets	1160 PM 13,826 non-PM	Various private hospital-based datasets	N	4 ophthalmologists 2 retinal experts	Colour fundus images	Various (Topcon, Canon, Zeiss, Kowa, Syseye)	512 × 512	META-PM
Liu 2010 [32]	Retrospective	Singapore	NS/80	20 PM 20 non-PM	Private hospital-based dataset (SCORM – Singapore)	20 PM 20 non-PM	Private hospital-based dataset (SCORM - Singapore)	N	1 ophthalmologist	Colour fundus images	NS	800 × 800	NS
Lu 2021a [27]	Mixed	China	33010/29645	5879 PM 26,131 non-PM	Various private hospital based datasets	1816 PM 5586 non-PM	Various private hospital based datasets	N	20 ophthalmologists (15 general ophthalmologists, 5 senior retinal specialists)	Colour fundus images	Various: Canon, NIDEK, Topcon and Zeiss	512 × 512	META-PM
Lu 2021b [29]	Retrospective	China	17330/13869	All myopic 12,65 PM 10,237 non-PM	Private hospital-based dataset (China)	All myopic 317 PM 2262 non-PM	Private hospital-based dataset (China)	N	20 ophthalmologists (15 general ophthalmologists, 5 senior retinal specialists)	Colour fundus images	Canon	512 × 512	META-PM
Tan 2021 [26]	Retrospective	Singapore	13325/25678	762 PM 12,058 non-PM	SEED and SNEC-HMC studies [59, 60]	1338 PM, 11,520 non-PM	Various private population-based and hospital-based datasets	N	Ophthalmologists Retinal specialists Non-medical professional graders (varies by set)	Colour fundus images	Training: CR-DGi with 10D SLR back Test: Various	400 × 500	META-PM [22]
Tang 2022 [34]	Retrospective	China	895/1395	851 PM 114 non-PM (training and validation)	Various private hospital-based datasets	**High consistency subgroup:** 210 PM 28 non-PM **Low consistency subgroup:** 112 PMs 80 non-PM	Various private hospital-based datasets	N	4 ophthalmologists	Colour fundus images	Kowa Nonmyd WX-3D or Canon CR-DGi	224 × 224	META-PM
Chen 2015^a^ [40]	Retrospective	Singapore	2258/NS	58 PM 2200 non-PM	SiMES [60]	NS	SiMES [60]	N	Clinical diagnosis from health records	Colour fundus images	NS (presumed as Zhang 2013)	NS	NS
Hemelings 2021^a^ [37]	Retrospective	Belgium	NS/1200	213 PM 187 non-PM	PALM [58]	NS	PALM [58], ODIR [59] and Messidor [57]	Y	7 ophthalmologists	Colour fundus images	Zeiss Visucam 500	288 × 288	NS
Himami 2022^a^ [36]	Retrospective	Indonesia	NS/612 306 PM 306 non-PM	**Method 1**: 550 Total **Method 2**: 488 Total (training and validation)	ODIR [59]	**Method 1**: 62 Total **Method 2**: 124 Total	ODIR [59]	Y	Expert ophthalmologists (unknown number) ^a^copied	Colour fundus images	Various: Canon, Zeiss, and Kowa cameras ^a^copied	512 × 512	META-PM
Li 2021a [63]	Retrospective	China	9316/35526	NS	EyePACS [56]	213 PM 187 non-PM	iChallenge PM [58]	Y	Training: self-supervised. Test: Health record data	Colour fundus images	Training: Zeiss VisuCam 500. Test: Various	320 × 320	NS
Rauf 2021^a^ [39]	Retrospective	Pakistan	NS/ 800	213 PM 187 non-PM	PALM [58]	NS	PALM [58]	Y	7 ophthalmologists	Colour fundus images	Zeiss Visucam 500	50 × 50	NS
Zhang 2013^a^ [38]	Retrospective	Singapore	2258/NS	58 PM 2200 non-PM	SiMES [60]	NS	SiMES [60]	N	Clinical diagnosis from health records	Colour fundus images	Canon CR-DGi with 10D DLR backing	384 × 256	NS

*NS* not stated.

^a^Studies excluded from the meta-analysis as no contingency table could be formed.

^b^From 4432 eyes.

**Table 2:** Model type and pooled performance for included studies.

Study	Model type	Model subtype	Hyper- parameters reported?	Evaluation method	Prevalence of pathological myopia in training dataset (%)	Prevalence of pathological myopia in test dataset (%)	Validation type	Sensitivity	Specificity	AUC (95% CI)	PPV (post-test probability)	NPV	LR−	LR+
Cen 2021 [35]	CNN	Various (Inception-V3, Xception, InceptionResNet-V2)	Yes	Held out test set + multiple external validation sets	5945/123,319	4768/111,230 (4.29%)	Internal and external^b^	0.9910	0.9963	Not estimable	0.9229	0.9996	0.0091	267.0934
Demir 2021 [25]	SVM/CNN	R-CNN + LSTM + SVM	Yes	10-fold cross validation	242/6426 (3.77SS%)	242/6426 (3.77%)	Internal	0.8802	0.9997	Not estimable	0.9907	0.9953	0.1199	2721.4711
Du 2021 [30]	CNN	EfficientNet (Keras)	Yes	Held-out test set + external validation	NS	981/1844 (53.20%)	Internal and external	0.8879	0.9583	Not estimable	0.9603	0.8826	0.1170	21.2842
Guo 2021 [28]	CNN	MobileNetV2 (lightweight CNN)	Yes	Held-out test set	40/202	9/48 (18.75%)	Internal	1.0000	1.0000	Not estimable	1.0000	1.00000	0.0000	N/A
Li 2021b [31]	CNN	Late-fusion multilabel model (SeResNext50)	Yes	Held-out test set + two external validation sets	8243/46,501 (17.7%)	389/8176 (4.76%)	Internal and external	0.9666	0.9601	Internal test set = 0.958 (0.952–0.965) External test set A = 0.992 (0.989–0.995) External test set B = 0.989 (0.988–0.991)	0.5473	0.9983	0.0348	24.2018
Li 2022 [33]	CNN	Dual-stream-based CNN (EfficientNte-B0 backbone)	No	Held-out test set + two external test sets	4862/40,594 (training and internal validation). 32475 in training set (approx.)	1160/14,986 (7.74%)	Internal and external	0.9198	0.9913	Internal validation = 0.997 (0.885–0.998) External validation = 0.998 (0.997–0.999), 0.994 (0.992–0.995)	0.8989	0.9933	0.0809	105.9795
Liu 2010 [32]	SVM	Proprietary method	No	Held-out test set with cross-validation	20/40 (50.0%)	20/40 (50.0%)	Internal	0.8500	0.9000	Not estimable	0.8947	0.8571	0.1667	8.5000
Lu 2021a [27]	CNN	ResNet18	No	5-fold cross validation + external validation	1250/6402 (19.5%)	1816/7402 (24.53%)	Internal and external	0.9466	0.9783	Cross validation = 0.995 (0.993–0.996) External validation = 0.989 (0.986–0.991)	0.9342	0.9826	0.0546	43.6994
Lu 2021b [29]	CNN	Xception	No	Held-out test set + external validation	1265/11,502	317/2579 (12.29%)	Internal and external	0.9432	0.9757	Primary test set = 0.993 (0.989–0.997) External validation = 0.989 (0.983–0.994)	0.8446	0.9919	0.0582	38.7920
Tan 2021 [26]	CNN	ResNet101 + multi-instance multiscale CNNs	No	Held-out test set	762/12,820 (5.94%)	1338/12,858 (10.41%)	Internal and external	0.9604	0.9098	Primary validation = 0·975 (0·970–0·981 External validation set 1 = 0.969 (0.959–0.977) External validation set 2 = 0·972 (0·938–0·994) External validation set 3 = 0·988 (0·980–0·994)	0.5529	0.9950	0.0435	10.6484
Tang 2022 [34]	CNN	ResNet-50	Yes	Held-out test set with 5-fold cross-validation	368/727 (50.61%)	120/238 (50.42%)	Internal	0.9667	0.9915	0.9980 (0.995–1.000)	0.9915	0.9669	0.0336	114.0667
Chen 2015^a^ [40]	Joint sparse multi-task learning	Joint sparse multi-task learning	No (only for comparators)	Cross-validation	58/2258	NS/2258	Internal	-	-	0.94 (NS)	-	-	-	-
Hemelings 2021^a^ [37]	CNN	UNet++/ResNet-18	Yes	Internal validation + unlabelled test set	213/400	NS/3750	Internal and external	-	-	0.9867 (NS)	-	-	-	-
Himami 2022^a^ [36]	CNN	ResNet-50 DenseNet-201	Yes	Held-out test set	NS/62	NS/426	Internal	0.9350	1.0000	Not estimable	-	-	-	-
Li 2021a [63]	CNN	ResNet-18 + kNN classifier	Yes	5-fold cross validation	NS/35126	NS/400	Internal	0.9912	-	0.9912 (NS)	0.9927	-	-	-
Rauf 2021^a^ [39]	CNN	2C-128N-0D (Spyder)	Yes	Internal validation + unlabelled test set	239/400	NS/400	Internal	-	-	0.9845 (NS)	-	-	-	-
Zhang 2013^a^ [38]	SVM	Multiple Kernel Learning (MKM)	Yes	Cross-validation	58/2258	NS/2258	Internal	0.71	0.85	0.852 (SD 0.044)	-	-	-	-

*AUROC* area under receiver-operator curve, *PPV* positive predictive value, *NPV* negative predictive value, *LR-* negative likelihood ratio, *LR+* positive likelihood ratio, *F1* F1-Score, *CNN* convolutional neural network, *SVM* support vector machine.

^a^Studies excluded from the meta-analysis as no contingency table could be formed from the published data.

^b^Only AUC and Accuracy were provided for external validation sets, hence these were excluded from the meta-analysis.

### Data analysis

2 × 2 contingency tables and statistics were generated using the best available data from study manuscripts (Table 1). Using the 2 × 2 contingency tables extracted for each study, sensitivity, specificity, positive predictive value, negative predictive value, negative likelihood ratio and positive likelihood ratio were calculated. Ungradable images were excluded from all analyses.

Where studies reported testing on both a primary, held-out test set and one or more external validation sets, the 2 × 2 contingency tables for each were combined to obtain a single contingency table. The combined contingency table was used in the meta-analysis. The contingency tables and model performance for the individual sets were recorded.

The presence of heterogeneity between included studies was assessed using the chi-square test and quantified by Higgins’ *I*^2^ [19], where significant heterogeneity was considered to be *I*^2^ ≥ 50%. The presence of threshold effects was assessed using the Spearman correlation coefficient between the logit of the true positive rate (TPR) and the false positive rate (FPR).

Where the heterogeneity among the included studies exceeded the stated threshold of 50%, measures were pooled using the DerSimonian and Laird (random-effects) model [20]. Where heterogeneity among the studies did not include the threshold, we planned to use a Mantel–Haenszel (fixed-effects) model [21].

Heterogeneity was investigated using subgroup analyses and meta-regression. Subgroup analyses were pre-specified in the study protocol, and included the type of model used and country of study/dataset origin. Sensitivity analyses were performed to assess the relationship between reviewer-assessed study quality and diagnostic accuracy and heterogeneity, in line with methods described by Higgins et al. [19]. Meta-regression was performed to analyse the relative effect of the size of the training set in each study.

The SROC curve was used to visually describe the relationship between the TPR and FPR in the included studies. The area under the SROC curve (AUROC) was calculated to demonstrate the probability of a classifier correctly classifying a randomly selected negative and positive example. Fagan nomograms were generated to describe the pre-test (prior) and post-test (posterior) probability for included studies, enabling direct translation of our results to the clinical setting.

Statistical analyses were performed using the Meta-Disc v1.4 software [22] and Review Manager 5.4 (The Cochrane Collaboration, 2020). Publication bias was assessed using the test described by Deeks et al. [23], implemented in R v4.1.3 using the *meta* package [24]. Fagan nomograms were generated in R v4.1.3 using the *TeachingDemos* package.

## Results

### Study selection

**Fig. 1:**
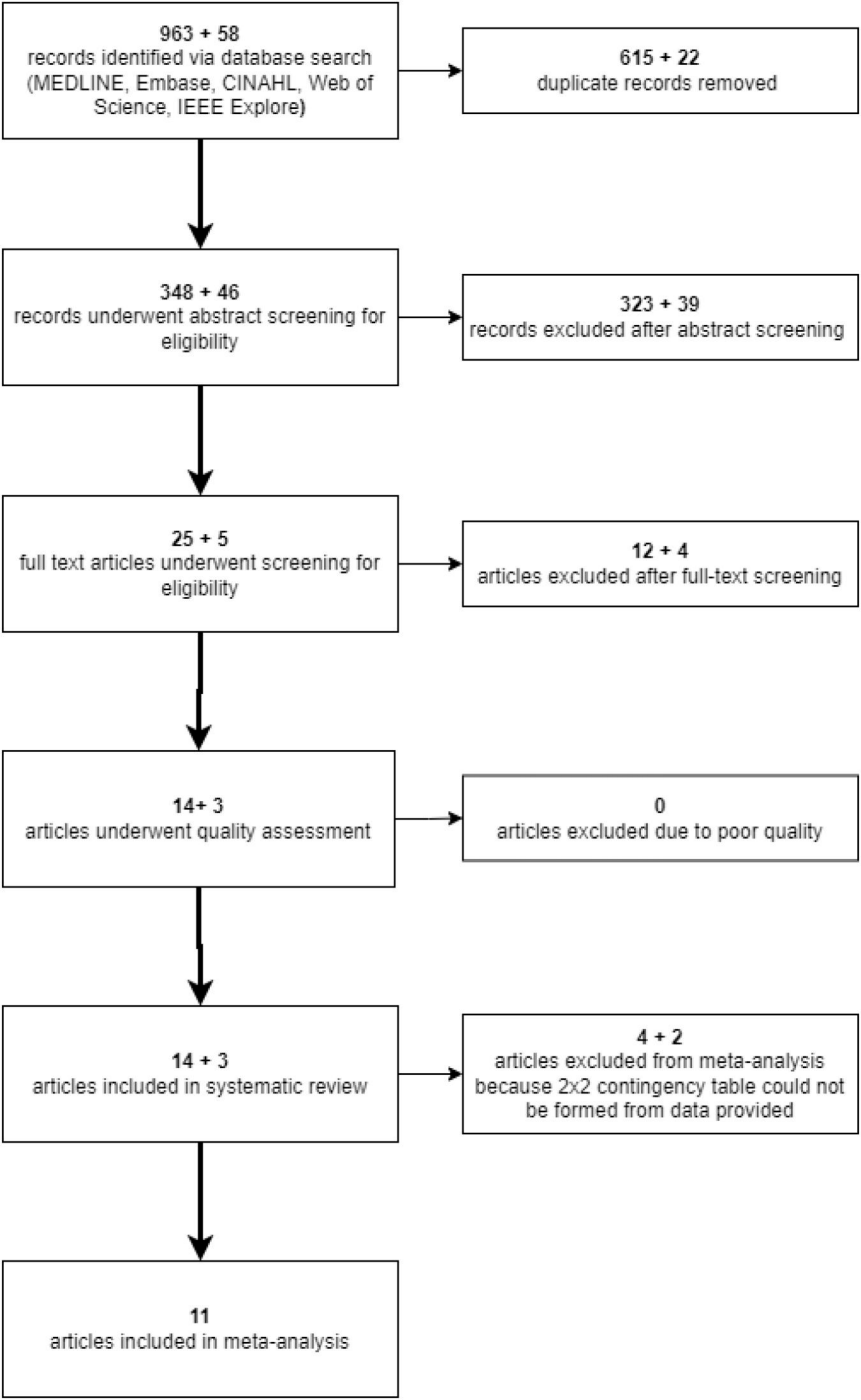
PRISMA flowchart showing study design. Added numbers (denoted with '+') represent studies added in the second database search.

Databases were initially searched for studies from inception to 20/01/22; searches were re-run on 05/05/22 to identify newly published studies. 1021 citations were identified via the database search. After duplicate removal, 394 citations underwent abstract screening for eligibility. Thirty full text articles were screened, and met the inclusion criteria (Fig. 1). Five studies resulted in reviewer disagreements at the abstract screening stage, and are reported in Supplementary Table 7. There were no disagreements between reviewers at the full-text screening stage. Eleven studies were included in the meta-analysis [25–35], and a further six [35–40] in the systematic review.

### Study quality assessment and publication bias

**Fig. 2:**
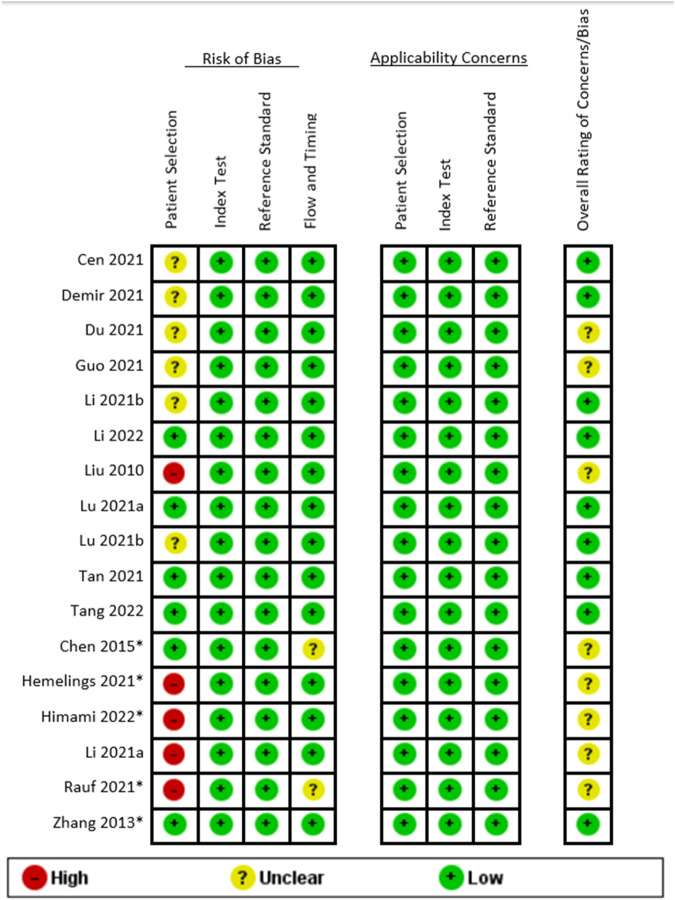
Results of QUADAS-2 quality assessment. Green = high quality; yellow = unclear; red = low quality.

The results of the quality assessment are reported in Fig. 2, and Supplementary Tables 2 and 3. The quality of included studies was fair, with all studies achieving either moderate or high quality. There were no disagreements between reviewers on the quality of included studies. No articles were excluded on the basis of poor quality.

Using the quantitative funnel plot test described by Deeks et al. [23], it was determined that publication bias was unlikely for studies included in the meta-analysis (*t* = −1.53, *p* = 0.1607). The qualitative funnel plot is shown in Supplementary Fig. 1.

### Study characteristics

All 17 included studies described and evaluated an AI-based method to identify pathological myopia from colour fundus images. Fourteen studies (82.4%) used convolutional neural network-based methods, with one of these studies also using a support vector machine (SVM) method and another using a k-nearest neighbours method for classification. Two studies (11.8%) [32, 38] used SVM for classification, and another [40] used joint sparse multi-task learning. Six studies (35.3%) used publicly available datasets, and two studies (11.8%) used some publicly available data. Demographic information, where reported, is presented in Supplementary Table 8. Eight studies (47.1%) used the META-PM definition for pathological myopia to guide annotation; the remainder did not use a formal definition. Eight studies (47.1%) included an external validation set. Seven studies (41.2%) compared the performance of the algorithm with that of one or more human graders, reported in Supplementary Table 10.

Fourteen studies (82.4%) used direct labelling by expert ophthalmologists or retinal specialists only as the reference standard. One study (5.9%) also used labelling by expert ophthalmologists and non-medical expert graders. One study (5.9%) used self-labelling methods for the training data and health record data for the test set. Two studies (11.8%) used health record data to generate labels.

### Performance of AI in detection of pathological myopia

**Fig. 3:**
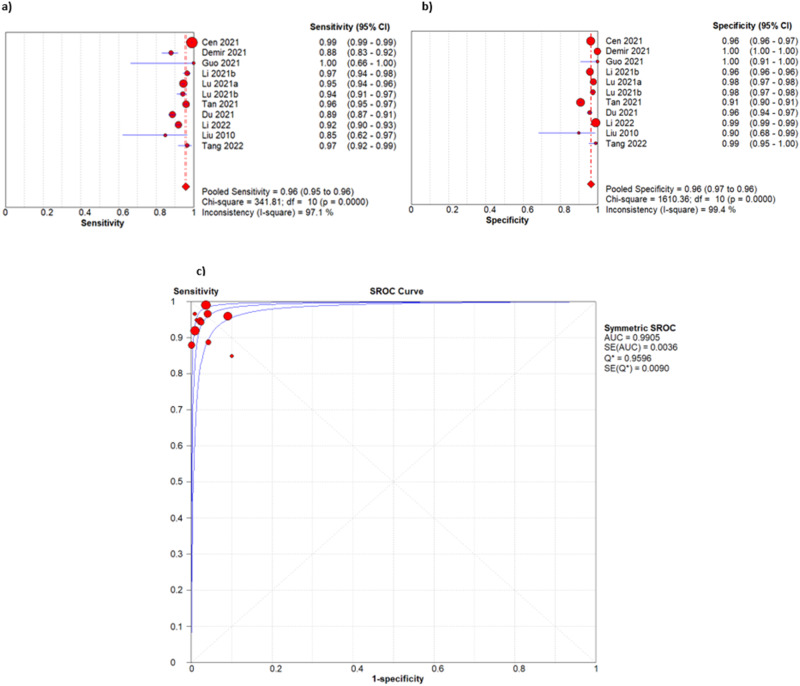
Forest plot and SROC curves for main analysis. Forest plot of sensitivities (**a**) and specificities (**b**) and SROC curve for overall diagnostic performance (**c**) for all studies included in meta-analysis. CI indicates confidence interval; SROC, Summary receiver operating characteristic, AUC indicates area under the curve, SE indicates standard error; Q* indicates where sensitivity = specificity (intersection of diagonal with SROC curve).

Eleven studies were included in the meta-analysis. The area under the SROC curve was 0.9905. The range of sensitivities reported was 0.850–1.000. The range of specificities reported was 0.900–1.000. All except three studies (27.3%) had a sensitivity and specificity above 0.900 [25, 30, 32]. The pooled sensitivity was 0.959 (95% CI 0.955–0.962, *I*^2^ 97.1%). The pooled specificity was 0.965 (95% CI 0.963–0.966, *I*^2^ 99.4%) (Fig. 3). Diagnostic odds ratios for included studies ranged from 51.00–22702.90 (Supplementary Table 6). The pooled diagnostic odds ratio (DOR) for detection of PM was 841.26 [95% CI 418.37–1691.61]. Fagan nomograms are used (Supplementary Fig. 4) to demonstrate the post-test probabilities of the included models, which ranged from 54.73% to 99.15%.

Individual contingency tables for included studies are reported in Supplementary Table 5.

### Performance comparison with human graders

7 of 11 studies included in the meta-analysis (63.6%) also reported a comparison with human graders, and are reported in Supplementary Table 10. Where reported, the mean sensitivities and specificities of the human graders ranged from 0.719–0.986 and 0.972–0.998, respectively. The corresponding proposed model sensitivities and specificities ranged from 0.908–0.991 and 0.925–1.000, respectively.

### Heterogeneity analysis

Heterogeneity across the included studies was substantial (Fig. 3). We sought to explain this heterogeneity using threshold analysis, meta-regression and subgroup analysis.

### Threshold analysis

A threshold analysis showed no significant effect (Spearman correlation coefficient = −0.112, *p* = 0.729), indicating that the heterogeneity observed between the included studies was unlikely to be due to a threshold effect.

### Subgroup and sensitivity analyses

We performed subgroup analyses as pre-specified in the study protocol. Subgroup analysis by publication year was deemed inappropriate due to the recent publication date of all except one study.

A subgroup analysis by classification algorithm was performed. Two studies [25, 31] used an SVM-based model for classification and were considered as a separate subgroup to studies using CNNs. Fifteen studies used a CNN-based approach. Although the two non-CNN studies had a significantly lower pooled sensitivity (0.878 [95% CI 0.832–0.915], *I*^2^ = *0.0%*) than the CNN studies (0.960 [95% CI 0.957–0.964], *I*^2^ = *97.4%*), the pooled specificity was higher (0.999 [95% CI 0.998–1.000], *I*^2^ = *94.3%*) than that of the CNN studies (0.961 [95% CI 0.959–0.962], *I*^2^ = *99.3%*). The AUROC for the CNN studies was 0.9925; as there were only two non-CNN studies, AUROC was not estimable for this subgroup. Subgroup analysis by study country (studies originating from China vs. other regions) and showed lower sensitivities, specificities and AUROC for studies originating outside China (Supplementary Figs. 2 and 3). Heterogeneity generally remained high (*I*^2^ > 90%) in subgroups, limiting interpretation.

Sensitivity analyses based on reviewer-assessed study quality using QUADAS-2 (Fig. 2) showed a significantly lower AUROC (0.859), sensitivity (0.888 [95% CI 0.867–0.907], *I*^2^ = *16.8%*) and specificity (0.959 [95% CI 0.944–0.971], *I*^2^ = *56.1%*) for studies with a ‘moderate’ risk of bias than sensitivity and specificity for studies with a ‘low’ risk of bias (0.993, 0.966 [95% CI 0.962–0.969], *I*^2^ = *97.1%*, 0.965 [95% CI 0.966–0.963], *I*^2^ = *99.6%*).

Similarly, sensitivity was higher in studies with an external validation group (0.960 [95% CI 0.956–0.964], *I*^2^ = 98.1%) versus without (0.908 [95% CI 0.875–0.935], *I*^2^ = 72.5%) (Table 2). However, specificity was higher in studies without an external validation group (0.999 [95% CI 0.998–1.000], *I*^2^ = 85.6%, vs 0.961 [95% CI 0.959–0.962], *I*^2^ = 99.5%)

### Meta-regression

Univariate meta-regression (Table 2) showed no statistically significant effect of training set size to account for the observed heterogeneity.

## Discussion

To our knowledge, this is the first systematic review and meta-analysis to show that AI-based screening methods are highly sensitive and specific for the diagnosis of pathological myopia from fundus images. The area under the SROC curve was 0.9905, suggesting excellent classification performance of included models, as well as high diagnostic odds ratios (pooled DOR = 841.26) suggesting that included models generally possessed robust discriminative ability. However, the presence of unexplained statistical heterogeneity means that results should be interpreted with caution.

The majority of included studies used CNN-based models to detect PM. Three of the studies reported lower sensitivities [25, 30, 31], two of which used SVM to classify images, which would be expected to have lower discriminative ability than a deep learning-based approach. While one study employing SVM [37] was excluded from the quantitative analysis, it also showed a lower discriminative ability relative to CNN-based models.

Fagan nomograms were used to describe the likelihood of a patient having PM if the diagnostic tool deemed them to be a positive case (post-test probability); these demonstrated generally high (>85%) post-test probabilities, suggesting that diagnostic decisions made by AI-based tools may offer clinicians a high degree of clinical certainty.

The potential application of AI models to diagnose PM in practice is multi-fold. Firstly, these analyses show a universally high observed diagnostic accuracy of AI-based models, reinforcing their capability as powerful screening tools for PM.

Secondly, our analyses reveal that the diagnostic accuracy and discriminative capability of these models is comparable to that provided by ophthalmologists, highlighting their capability as decision aids. In all seven studies comparing algorithm and grader performance, algorithm sensitivity and specificity was comparable with that of human graders, offering support for the use of such algorithms as screening or triage tools. The use of AI in an assistive capacity may reduce uncertainty in diagnosis and reduce the variability in the diagnosis made between healthcare professionals [41].

Tools based on AI models can work with clinicians to guide triage and referral decisions in general practice or non-specialist centres with a high case burden, as described by De Fauw et al. [42]. This may have particular benefit for clinicians in training, or in regions with reduced incidence of PM, who may derive benefit from decision support in selecting cases of PM [43], or in areas with poor access to healthcare services [44].

Identification and close follow-up of patients with uncomplicated PM is crucial in enabling early management of treatable complications such as myopic CNV—for example with anti-VEGF therapy [45]—optimisation of visual acuity and stabilisation of progressive myopia. As novel treatments, such as stem cell therapy, gain prominence in the management of retinal disease [46], timely and targeted intervention is likely to be effective in reducing the public health burden of PM. Identification of PM can enable prognostication and careful multidisciplinary planning to mitigate the social, economic [47] and cognitive [48] impacts of progressive visual loss on the individual.

No included studies reported on the implementation of AI-based screening methods in clinical practice. While this review highlights the potential of AI to make highly specific and sensitive judgements on the presence or absence of pathological myopia, consideration must be given to generalisability across populations, explainability of screening decisions [49], and patient and healthcare professional acceptability [50].

Several existing reviews and meta-analyses examine the sensitivity and specificity of AI-based methods for the detection of other ophthalmic conditions from fundus photographs. Dong et al. [12] performed a systematic review and meta-analysis of AI algorithms used for the diagnosis of age-related macular degeneration, finding a pooled sensitivity and specificity of 0.88 and 0.90 respectively. Chaurasia et al. [13] demonstrated a pooled sensitivity and specificity of 0.92 and 0.94 respectively for the diagnosis of glaucoma from fundus images using AI algorithms. Finally, a meta-analysis by Wu et al. [51] observed a combined AUROC of 0.97–0.99 for the use of AI in diabetic retinopathy screening.

Examples are present in the literature of the use of AI for the detection of PM from optical coherence tomography (OCT) images [32, 52]. However, this was beyond the scope of this study. At the time of writing, OCT machines remain expensive, rendering them inaccessible in many regions. Fundus imaging, however, is widespread, and screening tools based on fundus photography may have a more significant clinical impact in less economically developed regions.

Future research assessing the diagnostic performance of AI models using OCT images for detection of PM may be useful in regions where the technology is widely used.

### Strengths and weaknesses

The present study has several strengths. We used robust meta-analytic methodology to assess the pooled diagnostic accuracy of included studies, according to the PRISMA guidelines. Rigorous risk of bias assessment was performed, using two checklists, to identify studies which did not meet quality standards for inclusion, and heterogeneity and publication bias were comprehensively assessed using established methods.

Several limitations to this analysis are noted. First, there was significant statistical heterogeneity between the included studies, which was not entirely explained by analysis of threshold effects, study origin, training set size, study quality, the presence of external validation or the algorithm used.

A sensitivity analysis showed that studies deemed to have a moderate risk of bias reported a lower sensitivity and specificity, and studies without an external validation set reported a lower sensitivity—explaining some of the observed heterogeneity. Notably, there was considerable variation in the case-mix of positive to negative cases between studies, with PM prevalence varying from 3.78% in the test set used by Demir et al. to over 50% in other studies, potentially contributing to spectrum bias (where the discriminative ability of a diagnostic test varies according to the population in which it is used).

Meta-analyses of diagnostic accuracy of AI-based tools for the diagnosis of other ophthalmic conditions also demonstrate high unexplained heterogeneity [53], suggesting that variation in study and model design may have contributed to heterogeneity. However, it was not possible to assess the effects of variation in study design in detail (beyond subgroup analysis based on model type) due to the limited amount of methodological data provided in some studies. Regardless, the included studies spanned a diverse range of techniques and approaches, which—in the context of universally high diagnostic accuracy—suggests that AI-based techniques may possess excellent external validity for this purpose.

Secondly, several studies did not include an external validation set, limiting the generalisation ability of the algorithms reported. Ophthalmic patient populations are diverse, underlining the need for external validation, and meaning that the results of these studies should be interpreted with caution.

Third, there is a small degree of overlap between images contained within public datasets used in the studies, which could result in inflated estimates of diagnostic accuracy when comparing studies. Fourth, a retrospective study design was employed by all included studies. Retrospective design can lead to a selection bias, and have been shown to lead to overestimation of diagnostic accuracy [54]. Fifth, there was a preponderance of studies from East Asian countries, particularly China, in this review and meta-analysis. Planned subgroup analysis assessing studies by country of origin showed a small increase in sensitivity and specificity for studies originating from China, however it is unknown whether this is due to model factors or ethnic differences in fundus appearance [55].

Finally, due to the limitations of the available data, we did not include an analysis of model performance by severity of pathological myopia. It is therefore unknown how disease severity affects diagnostic accuracy in this case. Evidence suggests that use of severe cases may result in somewhat inflated estimates of diagnostic accuracy, and this would be an appropriate area for future research [54].

## Conclusion

This systematic review and meta-analysis provide robust early evidence for the diagnostic accuracy of AI-based tools in the diagnosis of PM. Such tools are likely to have significant impact in screening, triage, assisted diagnosis and monitoring of myopic patients, and may enable earlier diagnosis and improve clinical outcomes for patients at risk of developing PM.

## Summary

### What was known before

- Pathological myopia (PM) is an increasingly prevalent sight-threatening complication of high myopia, which requires close follow-up once identified to mitigate visual loss.
- The identification of PM from fundus images generally relies upon qualitative diagnosis by a healthcare professional.
- Artificial intelligence-based diagnostic tools have shown promise in ophthalmic diagnosis, but have not been specifically validated for use in PM.

### What this study adds

- Artificial intelligence-based algorithms are highly sensitive and specific for the diagnosis of PM from colour fundus images.
- These tools may hold potential for use in resource-constrained healthcare settings with a high prevalence of PM.

### Supplementary information

Supplementary Material

## Data Availability

All data analysed within this study was obtained from publicly available studies and/or public anonymised datasets. Figures and graphs were generated using freely available software packages. All studies, datasets and software packages used are referenced in full within the study and the Supplementary Material.
