# Supplementary material for: Performance of artificial intelligence for the detection of pathological myopia from colour fundus images: a systematic review and meta-analysis
Source: Eye (Lond). 2023 Aug 7;38(2):303–14. doi: 10.1038/s41433-023-02680-z (PMC10810874; doi:10.1038/s41433-023-02680-z)
Supplement: Supplementary file 1 — Supplementary Material [file 41433_2023_2680_MOESM1_ESM.pdf]

Diagnostic performance of artificial intelligence for the detection of pathological myopia from colour fundus images: a systematic review and meta-analysis

Supplementary Material

Table of Contents

Supplementary Table 1 - Representative search strategy (MEDLINE)

Supplementary Table 2 - Quality assessment of included studies (QUADAS-2)

Supplementary Table 3 – Quality assessment of included studies (CLAIM)

Supplementary Table 4 – Model performance by data set

Supplementary Table 5 – Contingency tables for included studies

Supplementary Table 6 – Diagnostic odds ratios (DORs) for included studies

Supplementary Table 7 – Reviewer screening conflicts and final decisions

Supplementary Table 8 – Patient demographics

Supplementary Table 9 – Results of threshold analysis

Supplementary Table 10 - Performance comparison with human graders

Supplementary Table 11 – Results of Sensitivity Analyses

Supplementary Figure 1 - Deeks' funnel plot for studies included in meta-analysis

Supplementary Figure 2 – Forest plots showing sensitivity and specificity for subgroup analyses

Supplementary Figure 3 – SROC curves for subgroup analyses

Supplementary Figure 4 – Fagan's nomograms for studies included in meta-analysis

Supplementary Table 1: Representative search strategy (MEDLINE)

exp Artificial Intelligence/	134232
('machine learning' OR 'artificial intelligence' OR 'AI' OR 'deep learning').tw.	72656
(automat* adj3 diagnos*).tw.	2581
(automat* adj3 detect*).tw.	11550
exp Myopia, Degenerative/	1520
('degenerat* adj5 myop*' OR 'myop* adj5 maculopathy' OR pathologic* adj5 myop*).tw.	1183
myop*.tw.	49900
1 or 2 or 3 or 4	178717
5 or 6 or 7	49980
8 and 9	**161**

Supplementary Table 2: Quality assessment of included studies (QUADAS-2)

Study	Patient selection			Index test		Reference standard		Flow and timing				Risk of bias				Concerns regarding applicability			Overall Rating**
	1	2	3	4	5	6	7	8	9	10	11	12	13	14	15	16	17	18	
**Cen 2021**	U	Y	Y	Y	NA	Y	Y	Y	Y	Y	Y	U	N	N	N	N	N	N	High
**Demir 2021**	U	Y	Y	Y	NA	Y	Y	Y	Y	Y	Y	U	N	N	N	N	N	N	High
**Du 2021**	U	Y	U	Y	NA	Y	Y	Y	Y	Y	Y	U	N	U	N	N	N	N	Med
**Guo 2021**	U	U	U	Y	NA	Y	Y	Y	Y	Y	Y	U	N	N	N	N	N	N	Med
**Li 2021b**	Y	Y	Y	Y	NA	Y	Y	Y	Y	Y	Y	U	N	N	N	N	N	N	High
**Li 2022**	Y	Y	Y	Y	NA	Y	Y	Y	Y	Y	Y	N	N	N	N	N	N	N	High
**Liu 2010**	U	N	Y	Y	NA	Y	Y	Y	Y	Y	Y	Y	N	N	N	N	N	N	Med
**Lu 2021a**	Y	Y	Y	Y	NA	Y	Y	Y	Y	Y	Y	N	N	N	N	N	N	N	High
**Lu 2021b**	U	Y	Y	Y	NA	Y	Y	Y	Y	Y	Y	U	N	N	N	N	N	N	High
**Tan 2021**	Y	Y	Y	Y	NA	Y	Y	Y	Y	Y	Y	N	N	N	N	N	N	N	High
**Tang 2022**	Y	Y	Y	Y	NA	Y	Y	Y	Y	Y	Y	N	N	N	N	N	N	N	High
**Chen 2015***	U	Y	U	Y	NA	Y	Y	Y	Y	Y	U	U	N	N	U	N	N	N	Med
**Hemelings 2021***	U	N	Y	Y	NA	Y	Y	Y	Y	Y	Y	Y	N	N	N	N	N	N	Med
**Himami 2022***	U	N	U	Y	NA	Y	Y	Y	Y	Y	Y	Y	N	N	N	N	N	N	Med
**Li 2021a***	U	N	U	Y	NA	Y	Y	Y	Y	Y	Y	Y	N	N	N	N	N	N	Med
**Rauf 2021***	U	N	U	Y	NA	Y	Y	Y	Y	Y	U	Y	N	N	U	N	N	N	Med
**Zhang 2013***	Y	Y	Y	Y	NA	Y	Y	Y	Y	Y	Y	N	N	N	N	N	N	N	High

Y: Yes; N: No; NA: not applicable.

- Studies excluded from the meta-analysis as no contingency table could be formed.

1 – Was a consecutive or random sample of patients enrolled?
2 – Was a case-control design avoided?
3 – Did the study avoid inappropriate exclusions?

4 – Were the index results interpreted without knowledge of the results of the reference standard?
5 – If a threshold was used, was it pre-specified?

6 – Is the reference standard likely to correctly classify the target condition?
7 - Were the reference standard results interpreted without knowledge of the results of the index test?

8 - Was there an appropriate interval between index test(s) and reference standard?
9 - Did all patients receive a reference standard?
10 - Did all patients receive the same reference standard?
11 - Were all patients included in the analysis?

12 - Could the selection of patients have introduced bias?
13 - Could the conduct or interpretation of the index test have introduced bias?
14 - Could the reference standard, its conduct, or its interpretation have introduced bias?
15 - Could the patient flow have introduced bias?

16 - Are there concerns that the included patients do not match the review question?
17 - Are there concerns that the index test, its conduct, or interpretation differ from the review question?
18 - Are there concerns that the target condition as defined by the reference standard does not match the review question?

Supplementary Table 3: Quality Assessment of Included Studies (CLAIM)

Study	Manuscript				Design		Data							Ground Truth					Data Partitions			Model			Training			Evaluation					Results					Discussion		Others		
	1	2	3	4	5	6	7	8	9	10	11	12	13	14	15	16	17	18	19	20	21	22	23	24	25	26	27	28	29	30	31	32	33	34	35	36	37	38	39	40	41	42
**Cen 2021**	Y	Y	Y	Y	Y	Y	Y	N	Y	na	Y	Y	N	Y	na	Y	Y	Y	na	Y	Y	Y	U	Y	Y	na	Y	Y	Y	Y	N	Y	Y	Y	Y	N	Y	Y	N	N	N	N
**Demir 2021**	Y	Y	Y	Y	Y	N	Y	N	N	na	Y	N	N	Y	na	N	Y	Y	na	Y	Y	Y	Y	Y	Y	Y	Y	Y	N	Y	Y	N	U	N	Y	Y	Y	Y	Y	N	N	Y
**Du 2021**	Y	Y	Y	Y	Y	Y	Y	N	Y	na	Y	Y	na	Y	na	Y	Y	Y	na	Y	Y	Y	Y	Y	Y	na	Y	Y	Y	Y	N	Y	Y	Y	Y	Y	Y	Y	N	N	Y	na
**Guo 2021**	Y	Y	Y	Y	Y	Y	Y	N	N	N	Y	N	N	Y	na	Y	Y	Y	na	Y	Y	Y	Y	Y	Y	na	Y	Y	Y	Y	N	N	N	Y	Y	Y	Y	Y	N	N	Y	Y
**Li 2021b**	Y	Y	Y	Y	Y	Y	Y	Y	Y	Y	Y	Y	Y	Y	na	Y	Y	Y	na	Y	Y	Y	Y	Y	Y	Y	Y	Y	Y	Y	Y	Y	N	Y	Y	N	Y	Y	N	N	Y	Y
**Li 2022**	Y	Y	Y	Y	Y	Y	Y	N	Y	Y	Y	Y	N	Y	na	Y	Y	Y	na	Y	Y	U	Y	N	Y	na	Y	Y	Y	Y	N	Y	N	Y	Y	Y	Y	Y	N	N	Y	Y
**Liu 2010**	Y	Y	N	Y	Y	Y	Y	N	Y	na	Y	N	N	Y	na	Y	Y	Y	na	Y	Y	N	U	N	Y	na	N	N	Y	Y	N	Y	N	Y	N	N	N	Y	N	N	N	na
**Lu 2021a**	Y	Y	Y	Y	Y	Y	Y	N	Y	na	Y	Y	N	Y	na	Y	Y	Y	na	Y	Y	Y	Y	U	Y	Y	na	Y	Y	Y	Y	N	Y	Y	Y	Y	Y	Y	Y	N	N	Y
**Lu 2021b**	Y	Y	Y	Y	Y	Y	Y	N	Y	Y	Y	Y	N	Y	na	Y	Y	Y	na	Y	Y	Y	Y	U	N	Y	na	Y	Y	Y	Y	Y	Y	N	Y	Y	Y	Y	Y	N	N	Y
**Tan 2021**	Y	Y	Y	Y	Y	Y	Y	Y	N	Y	Y	Y	Y	Y	na	Y	Y	Y	na	Y	Y	Y	Y	Y	Y	Y	Y	Y	Y	Y	N	U	Y	Y	Y	N	Y	Y	N	N	Y	na
**Tang 2022**	Y	Y	Y	Y	Y	Y	Y	Y	Y	Y	Y	Y	N	Y	na	Y	Y	Y	na	Y	Y	U	Y	Y	Y	Y	Y	Y	Y	Y	Y	Y	Y	N	Y	Y	Y	Y	Y	N	N	Y
**Chen 2015***	Y	Y	Y	Y	Y	N	Y	N	N	N	Y	N	N	Y	na	U	Y	Y	na	Y	Y	N	U	N	Y	na	Y	N	N	Y	N	Y	N	Y	N	N	N	Y	N	N	N	na
**Hemelings 2021***	Y	Y	Y	Y	Y	Y	Y	N	Y	Y	Y	N	N	Y	na	Y	Y	Y	na	Y	Y	Y	Y	Y	Y	Y	na	Y	N	Y	Y	N	Y	N	Y	N	N	Y	Y	N	N	Y
**Himami 2022***	Y	Y	Y	Y	Y	N	Y	N	Y	na	Y	N	N	Y	na	U	Y	Y	na	Y	Y	U	Y	Y	Y	na	Y	N	Y	Y	N	N	N	Y	N	N	N	Y	N	N	N	Y
**Li 2021a***	Y	Y	Y	Y	Y	Y	Y	N	Y	na	Y	na	na	Y	na	N	N	N	na	Y	Y	Y	Y	Y	Y	Y	na	Y	N	Y	Y	N	N	N	Y	P	N	Y	Y	N	N	N

**Rauf 2021***	N	Y	Y	Y	Y	Y	Y	N	Y	na	Y	N	N	Y	na	Y	Y	Y	na	Y	Y	Y	U	Y	Y	na	Y	N	N	Y	N	Y	N	Y	N	N	N	Y	N	N	N	na
**Zhang 2013***	N	Y	Y	Y	Y	Y	Y	Y	Y	Y	Y	N	Y	Y	na	U	Y	Y	na	Y	Y	Y	U	Y	Y	na	Y	N	Y	N	Y	Y	N	Y	N	N	N	Y	N	N	Y	Y

Y = Yes; N = No; U = unclear; P = partially satisfied, n/a = not applicable.

1 – Identification as a study of AI methodology, specifying the category of technology used (e.g., deep learning)

2 – Structured summary of study design, methods, results, and conclusions

3 – Scientific and clinical background, including the intended use and clinical role of the AI approach

4 – Study objectives and hypotheses

5 – Prospective or retrospective study

6 – Study goal, such as model creation, exploratory study, feasibility study, non-inferiority trial

7 – Data sources

8 – Eligibility criteria: how, where, and when potentially eligible participants or studies were identified (e.g. symptoms, results from previous tests, inclusion in registry, patient-care setting, location, dates)

9 – Data pre-processing steps

10 – Selection of data subsets, if applicable

11 – Definitions of data elements, with references to Common Data Elements

12 – De-identification methods

13 – How missing data were handled

14 – Definition of ground truth reference standard, in sufficient detail to allow replication

15 – Rationale for choosing the reference standard (if alternatives exist)

16 – Source of ground-truth annotations; qualifications and preparation of annotators

17 – Annotation tools

18 – Measurement of inter- and intrarater variability; methods to mitigate variability and/or resolve discrepancies

19 – Intended sample size and how it was determined

20 – How data were assigned to partitions; specify proportions

21 – Level at which partitions are disjoint (e.g., image, study, patient, institution)

22 – Detailed description of model, including inputs, outputs, all intermediate layers and connections

23 – Software libraries, frameworks, and packages.

24 – Initialization of model parameters (e.g., randomization, transfer learning)

25 – Details of training approach, including data augmentation, hyperparameters, number of models trained

26 – Method of selecting the final model

27 – Ensembling techniques, if applicable

28 – Metrics of model performance

29 – Statistical measures of significance and uncertainty (e.g., confidence intervals)

30 – Robustness or sensitivity analysis

31 – Methods for explainability or interpretability (e.g., saliency maps), and how they were validated

32 – Validation or testing on external data

33 – Flow of participants or cases, using a diagram to indicate inclusion and exclusion

34 – Demographic and clinical characteristics of cases in each partition

35 – Performance metrics for optimal model(s) on all data partitions

36 – Estimates of diagnostic accuracy and their precision (such as 95% confidence intervals)

37 – Failure analysis of incorrectly classified cases

38 – Study limitations, including potential bias, statistical uncertainty, and generalizability

39 – Implications for practice, including the intended use and/or clinical role

40 - Registration number and name of registry

41 - Where the full study protocol can be accessed

42 - Sources of funding and other support; role of funders

* Studies excluded from the meta-analysis as no contingency table could be formed.

Supplementary Table 4: Model performance by data set

Study	Dataset	Sensitivity	Specificity	PPV	NPV	LR-	LR+
Cen 2021	Primary validation	0.9934	0.9985	0.9695	0.9997	0.0066	654.5413
	Primary test	0.9970	0.9955	0.8940	0.9999	0.0030	220.9783
	External (multihospital) test	0.9903	0.9959	0.9111	0.9996	0.0097	239.1708
	Public test	0.9577	0.9876	0.9903	0.9464	0.0428	77.0986
Demir 2021	Cross-validation	0.8802	0.9997	0.9907	0.9953	0.1199	2721.471
Du 2021	Primary test	0.8879	0.9583	0.9603	0.8826	0.1170	21.2842
Guo 2021	Primary test	1.0000	1.0000	1.0000	1.0000	0.0000	-
Li 2021b	Primary test	0.9583	0.9711	0.6525	0.9976	0.0429	33.1212
	External test set A	0.9524	0.9897	0.8696	0.9965	0.0481	92.1429
	External test set B	0.9912	0.9379	0.3578	0.9997	0.0094	15.9718
Li 2022	External validation set 1	0.9328	0.9960	0.9478	0.9948	0.0675	235.7489
	External validation set 2	0.9098	0.9870	0.8636	0.9918	0.0914	70.2181
Liu 2010							
	Primary test	0.8500	0.9000	0.8947	0.8571	0.1667	8.5000
Lu 2021a	Cross-validation	0.9392	0.9819	0.9266	0.9852	0.0619	52.0297
	External validation	0.9629	0.9355	0.9511	0.9508	0.0397	14.9249
Lu 2021b	Primary test	0.9722	0.9774	0.8413	0.9965	0.0284	43.0724
	External validation	0.9051	0.9725	0.8493	0.9836	0.0976	32.9131
Tan 2021	Primary validation	0.9140	0.9421	0.4595	0.9951	0.0913	15.7935
	External test set 1	0.9680	0.8733	0.1481	0.9992	0.0366	7.6411
	External test set 2	0.9841	0.8554	0.9827	0.8659	0.0186	6.8069
	External test set 3	0.9419	0.9592	0.8798	0.9811	0.0606	23.0616
Tang 2022	Primary test	0.9667	0.9915	0.9915	0.9669	0.0336	114.0667
Chen 2015*	Cross-validation	-	-	-	-	-	-

Hemelings 2021*	Internal validation set	-	-	-	-	-	-
	External validation set	-	-	-	-	-	-
Himami 2022*	Primary test	0.935	1.0000	-	-	-	-
Li 2021a*	Internal validation	0.9912	-	0.9927	-	-	-
Rauf 2021*	Internal validation set	-	-	-	-	-	-
	External validation set	-	-	-	-	-	-
Zhang 2013*	Cross-validation	0.71	0.85	-	-	-	-

* Studies excluded from the meta-analysis as no contingency table could be formed.

Supplementary Table 5: Contingency tables for included studies

Study	Sample	TP	FP	FN	TN
**Cen 2021**	**Pooled**	**4725**	**395**	**43**	**106067**
[redacted]	Primary validation	1049	33	7	21711
	Primary test	1012	120	3	26476
	Multihospital test	2460	240	24	57721
	Public test set	204	2	9	159
**Demir 2021**	**Cross-validation**	**213**	**2**	**29**	**6182**
**Du 2021**	Primary test	**871**	**36**	**110**	**827**
**Guo 2021**	Primary test	**9**	**0**	**0**	**39**
**Li 2021b**	**Pooled**	**376**	**311**	**13**	**7476**
[redacted]	Primary test	184	98	8	3289
	External test set A	80	12	4	1149
	External test set B	112	201	1	3038
**Li 2022**	**Pooled**	**1067**	**120**	**93**	**13706**
[redacted]	External validation set 1	472	26	34	6545
	External validation set 2	595	94	59	7161
**Liu 2010**	**Primary test**	17	2	3	18
**Lu 2021a**	**Pooled**	**1719**	**121**	**97**	**5465**
[redacted]	Cross-validation	1174	93	76	5059
	External validation	545	28	21	406
**Lu 2021b**	**Pooled**	**299**	**55**	**18**	**2207**
[redacted]	Primary test	175	33	5	1429
	External validation	124	22	13	778
**Tan 2021**	**Pooled**	**1285**	**1039**	**53**	**10481**
[redacted]	Primary validation	255	300	24	4884
	External validation set 1	121	696	4	4798
	External validation set 2	682	12	11	71
	External validation set 3	227	31	14	728
**Tang 2022**	**Primary test**	**116**	**1**	**4**	**117**
**Total**		**10680**	**2080**	**460**	**152567**

TP = true positives; FP = false positives; FN = false negatives; TN = true negatives.

Supplementary Table 6 – Diagnostic odds ratios (DORs) for included studies

Study	Diagnostic odds ratios (95% CI)
Cen 2021	2950.45 (2149.76-4049.36)
Demir 2021	22,702.86 (5382.18 - 95,764.08)
Du 2021	181.90 (123.38 - 268.17)
Guo 2021	1,501.00 (27.95 - 80,603.39)
Li 2021b	695.27 (395.39 - 1,222.59)
Li 2022	1,310.42 (992.53 - 1,730.13)
Liu 2010	51.00 (7.57 - 343.73)
Lu 2021a	800.40 (609.45 - 1,051.19)
Lu 2021b	666.56 (386.21 - 1,150.42)
Tan 2021	244.58 (184.47 - 324.26)
Tang 2022	3,393.00 (373.59 - 30,815.70)
**Random Effects Model**	
Pooled Diagnostic Odds Ratio =	841.26 (418.37 – 1,691.6)
Cochran-Q =	237.59; df = 11 (p = 0.0000)
Inconsistency (I-squared) =	95.7%
Tau-squared =	1.1177

CI; confidence interval.

Supplementary Table 7: Reviewer screening conflicts and final decisions

Citation	Title	Result	Justification
Foo et al. 2021	Is artificial intelligence a solution to the myopia pandemic?	Study excluded	Article was a review, did not contain machine learning algorithm or primary results
Lin et al. 2018	Prediction of myopia development among Chinese school-aged children using refraction data from electronic medical records: A retrospective, multicentre machine learning study.	Study excluded	Did not fit the inclusion criterion of colour fundus images.
Shi et al. 2021	Research on screening system of myopic maculopathy based on deep convolution neural network	Study excluded	Article satisfied all inclusion criteria, however main text was not written in English.
Sun et al. 2021	Imaging Features by Machine Learning for Quantification of Optic Disc Changes and Impact on Choroidal Thickness in Young Myopic Patients	Study excluded	Article did utilise colour fundus images and machine learning, however, did not have pathological myopia as a condition in sample, only high myopia.
Yang et al. 2020	Prediction of Myopia in Adolescents through Machine Learning Methods.	Study excluded	Article did utilise colour fundus images and machine learning, however, did not have pathological myopia as a condition in sample, only high myopia.

Supplementary Table 8: Patient demographics

Study	Demographic data	
	Age (years) (Mean [SD]; range)	*Sex (% Female)*
**Cen 2021**	Total Dataset: 51.7-61.3	Total Dataset: 50-61%
**Demir 2021**	NS	NS
**Du 2021**	Training & Validation Dataset: 65 [15.0]<br>Testing Dataset: 58 [19.0]	NS
**Guo 2021**	NS	NS
**Li 2021b**	Primary Test Dataset: 55.4 [18.3]; 2-96)<br>External Test Dataset A: 48.7 [18.0]; 4-89)<br>External Test Dataset B: 52.6 [20.6]; 3-97	NS
**Li 2022**	NS	NS
**Liu 2010**	NS	NS
**Lu 2021a**	Total Dataset: 43.11-63.22; 23-82<br>External Validation: 39.5-63.7; 16-81	Total Dataset: 54.5-71.1%<br>External Validation: 49.3-66.1%
**Lu 2021b**	Total Dataset: 48.8-61.3<br>External Validation: 49.8-59.0	Total Dataset: 55.8-68.9%<br>External Validation: 55.8-66.2%
**Tan 2021**	Primary & Validation Dataset: 57.5 [10.1]	Primary & Validation Dataset: 52.1%
**Chen 2015***	NS	NS

**Hemelings 2021***	NS	NS
**Rauf 2021***	NS	NS
**Li 2021a**	NS	NS
**Zhang 2013***	NS	NS

* Studies excluded from the meta-analysis as no contingency table could be formed.

<u>Supplementary Table 9: Results of threshold analysis</u>

**Spearman correlation coefficient**	-0.064
**Spearman *p***	0.853

Supplementary Table 10: Performance comparison with human graders

Study	Description of comparison/subset used for comparison	Type of grader used	Mean sensitivity of human graders	Sensitivity of proposed model	Mean specificity of human graders	Specificity of proposed model	Mean accuracy of human graders	Accuracy of proposed model
**Cen 2021**	External comparative dataset of 922 images. Each specialist labelled all images.	5 retinal specialists	0.952	0.984	0.998	1.000	NS	NS
**Du 2021**	**Graders**: 400 randomly selected images consisting of 100 images per category from the test set<br>**Model**: 1844 images (test set)	1 trained grader and 1 myopia specialist	NS	NS	NS	NS	0.939	0.921
**Li 2021b**	External test set B (3352 images). Each resident labelled a quarter of the test B (838 images).	4 ophthalmologist residents	0.719	0.991 (0.989-0.993) *	0.992	0.938 (0.934-0.943) *	NS	NS
**Li 2022**	Random sample of 3000 images from two external testing sets. All graders labelled all images independently.	2 senior ophthalmologists (>5 years' experience) and 2 junior ophthalmologists (<2 years' experience)	0.927	0.908	0.972	0.991	0.927	0.908
**Lu 2021a**	External validation dataset only (1000 images)	1 ophthalmologist<br>1 retinal specialist	0.956	0.963 (0.951-0.974)	0.979	0.935 (0.920-0.950)	0.969	0.951
**Lu 2021b**	Test set for binary task only – 1642 images	1 ophthalmologist<br>1 retinal specialist	0.986	0.977 (0.970–0.985)	0.978	0.972 (0.962–0.980)	0.985	0.977
**Tan 2021**	"200 images with and without myopic macular degeneration (in a 1:4 ratio) were randomly selected using a random number generator from the primary validation dataset, and external test datasets 1, 3, and 4"	Six human experts (five retinal specialists and one ophthalmologist)	0.721	0.975 (0.917 – 1.000)	0.978	0.925 (0.885 – 0.962)	0.927	0.935

***whole of external test set B**

Supplementary Table 11: Results of sensitivity analyses

	AUROC		Sensitivity			Specificity		
**Risk of bias**	**AUROC**	**Standard Error (SE)**	**Sensitivity**	**95% Confidence Interval**	**I^2 (Heterogeneity)**	**Specificity**	**95% Confidence Interval**	**I^2 (Heterogeneity)**
Low	0.993	0.002	0.966	0.962-0.969	97.1%	0.965	0.966-0.963	99.6%
Moderate	0.859	0.143	0.888	0.867-0.907	16.8%	0.959	0.944-0.971	56.1%
**External validation**								
With external validation dataset	0.992	0.003	0.960	0.956-0.964	98.1%	0.961	0.959-0.962	99.5%
Without external validation dataset	0.977	0.025	0.908	0.875-0.935	72.5%	0.999	0.998-1.000	85.6%

Supplementary Figure 1: Deeks' funnel plot for studies included in meta-analysis.

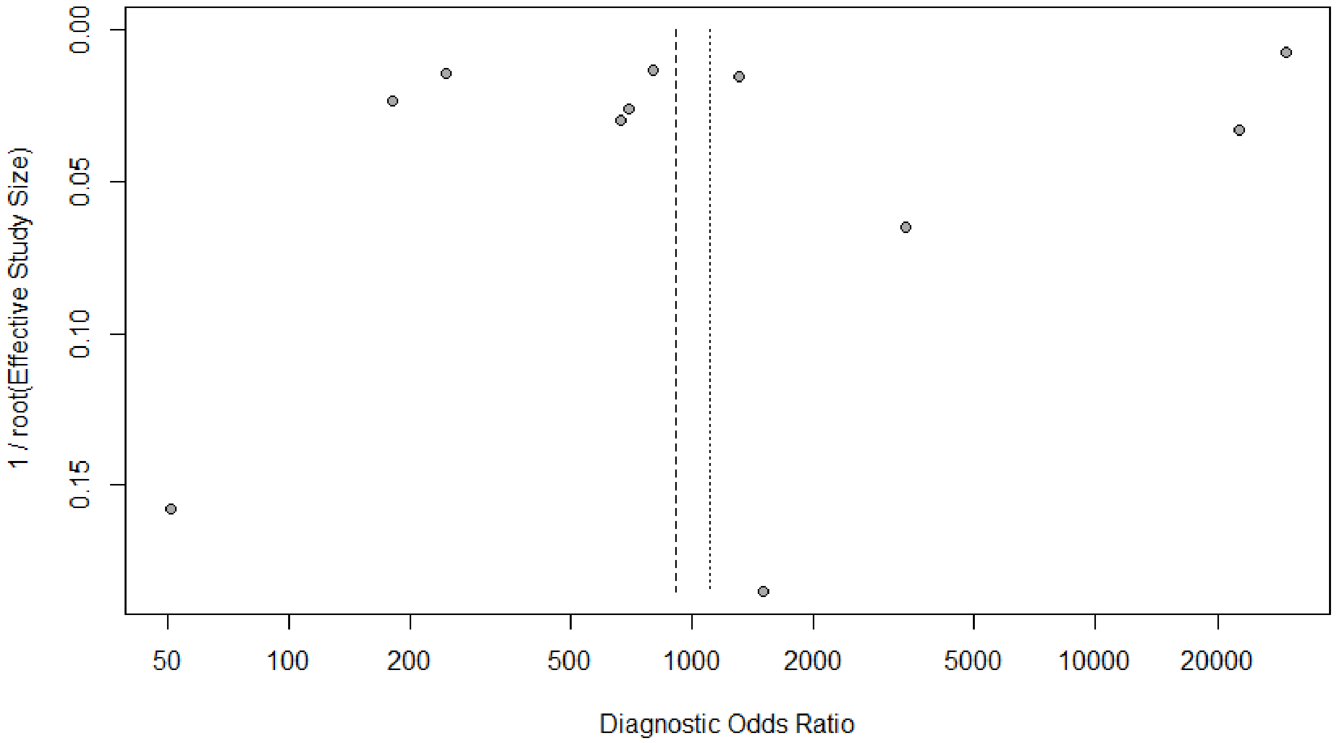

Deeks' test: t = -1.53, p = 0.1607

<u>**Supplementary Figure 2 – Forest plots showing sensitivity and specificity for subgroup analyses**</u>

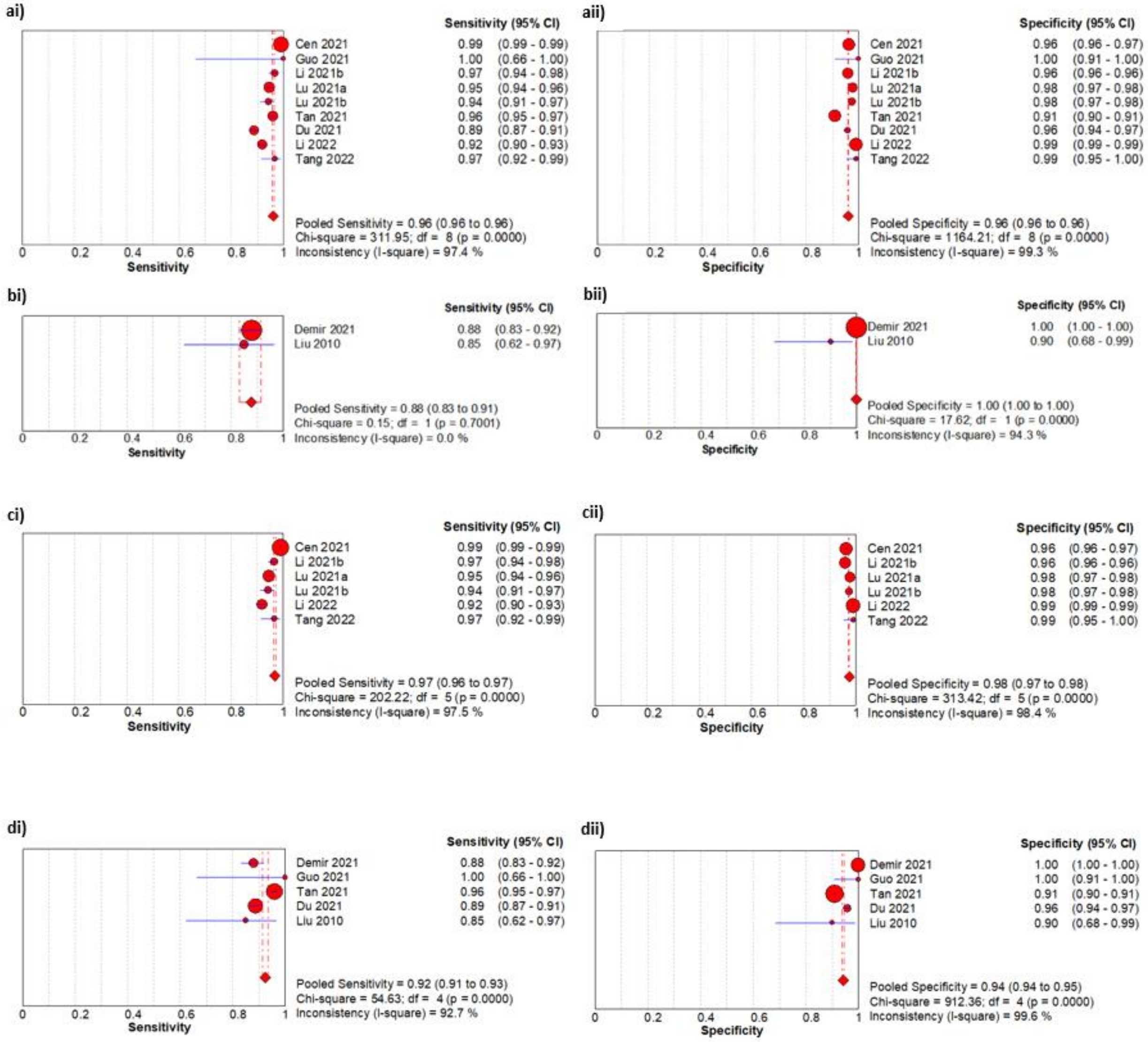

Supplementary Figure 2. Forest plots for sensitivity and specificity analysis of overall diagnostic performance in: ai&aii) CNN studies, bi&bii) Non-CNN studies, ci&cii) Studies originating from China and di&dii) Studies originating from outside China included in the meta-analysis. CI = confidence interval.

Supplementary Figure 3 – SROC curves for subgroup analyses

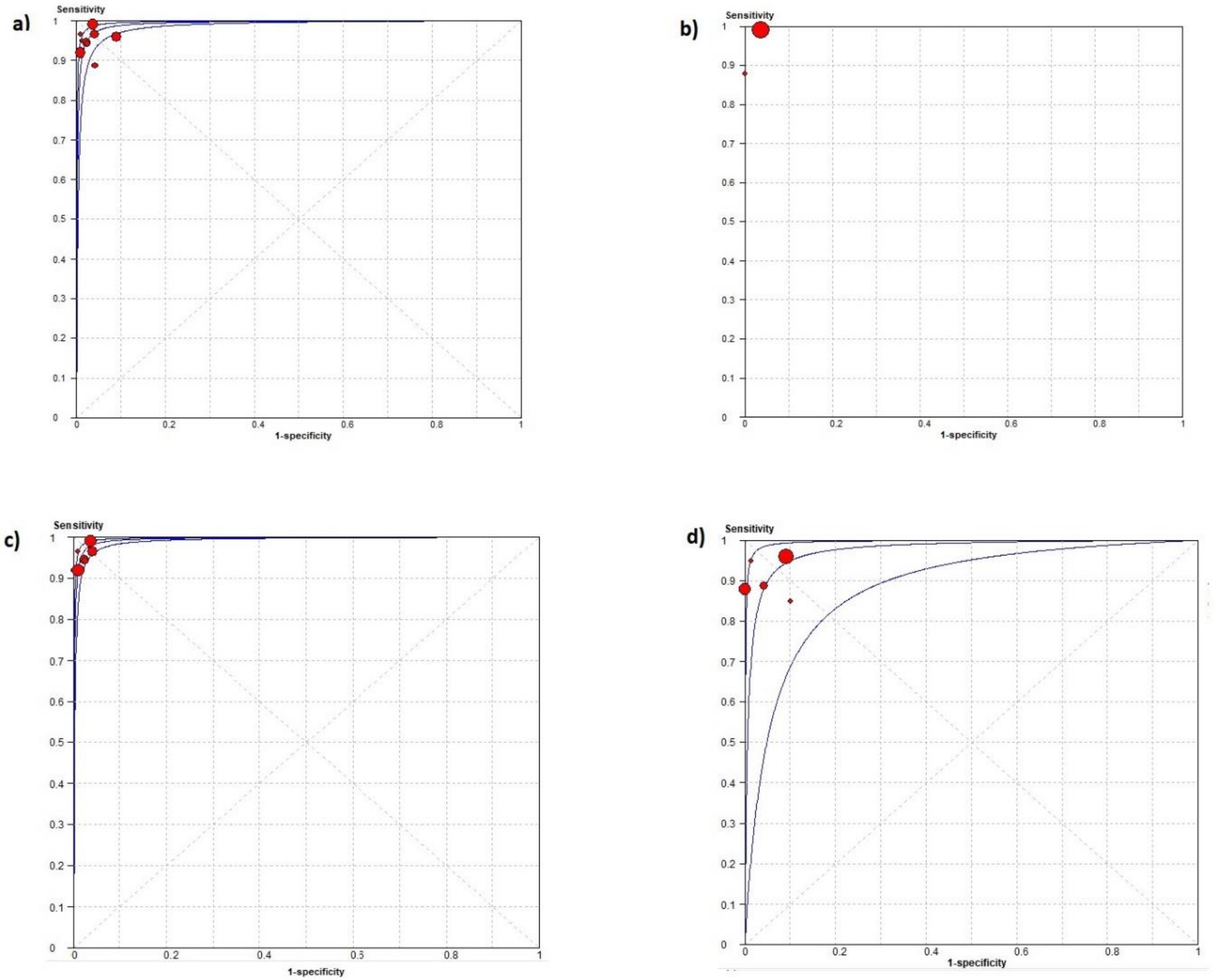

Supplementary Figure 3. SROC curves for overall diagnostic performance in: a) studies using CNN, b) studies not using CNN, c) studies originating from China, d) studies originating from outside China included in the meta-analysis. SROC, Summary receiver operating characteristic; CNN, convolutional neural network. *AUC (SE)*: a) 0.9925 (0.0024), b) not estimable due to small number of studies, c) 0.9951 (0.0012),d) 0.9751 (0.0129).

Supplementary Figure 4 – Fagan's nomograms for studies included in the meta-analysis

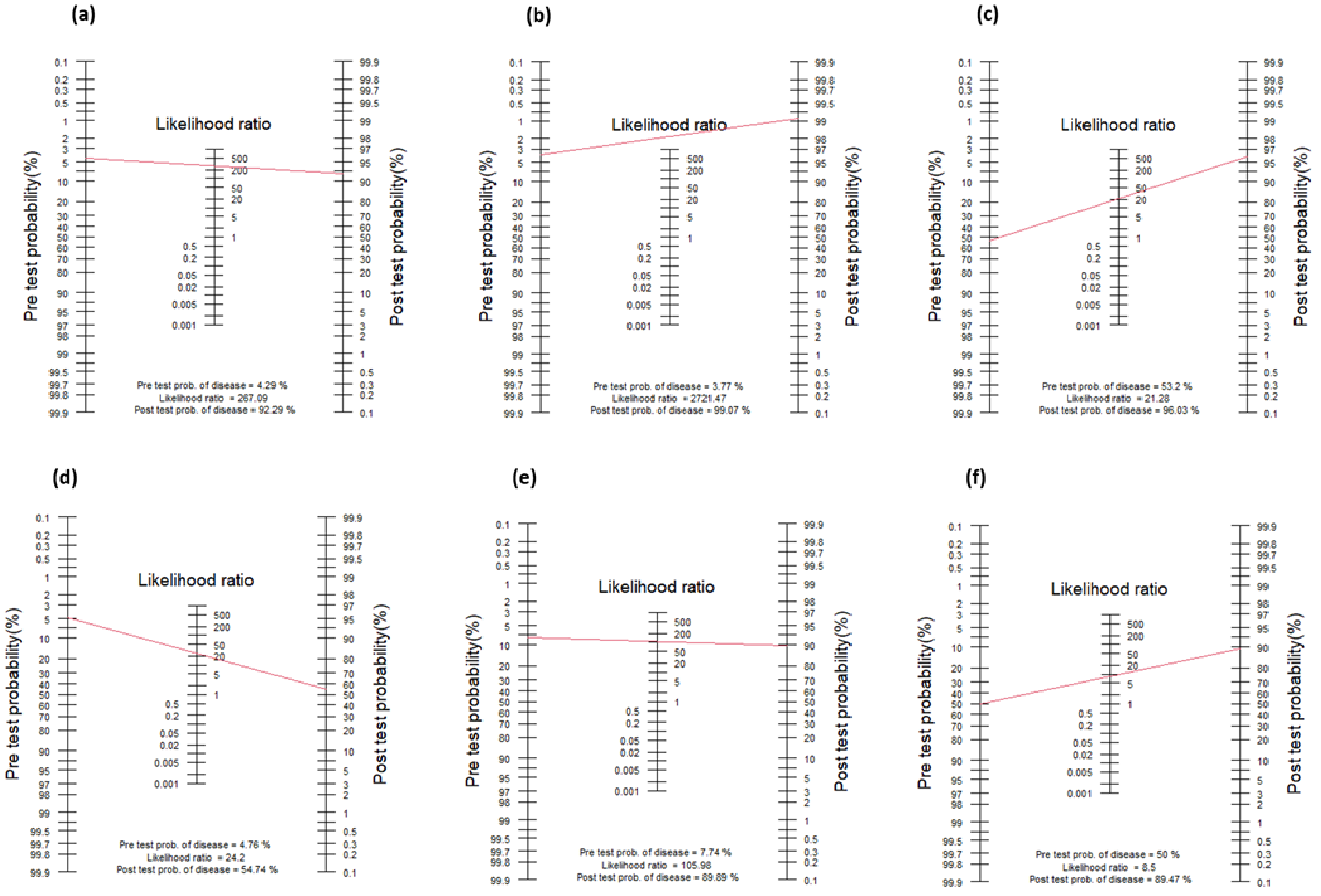

(g) (h) (i)

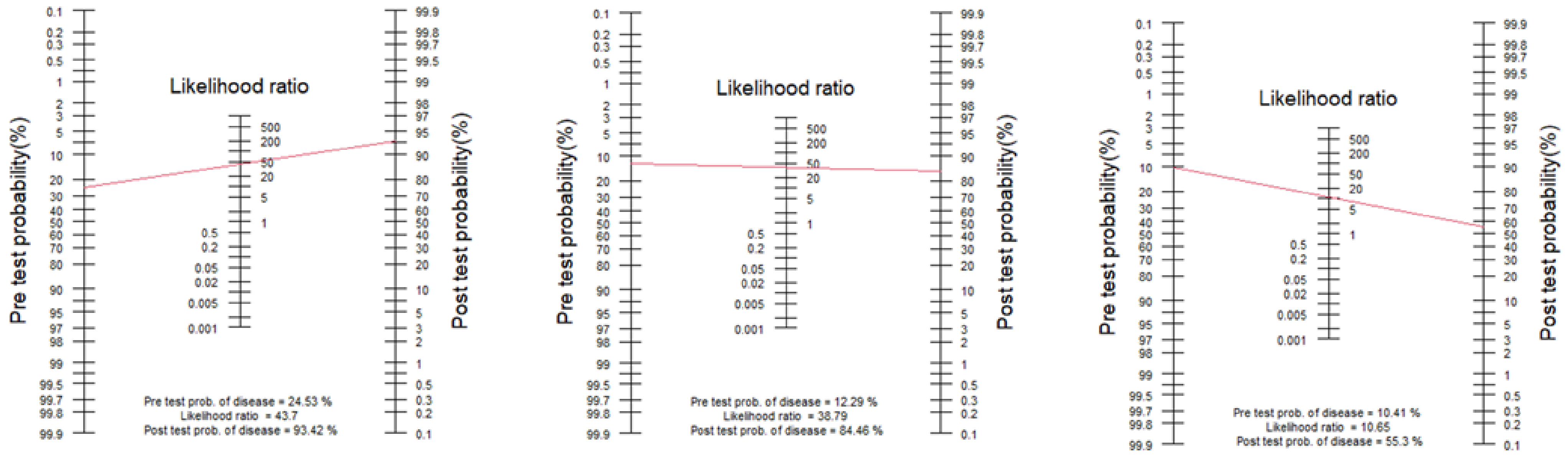
Pre test probability(%)
Likelihood ratio
Post test probability(%)
Pre test prob. of disease = 24.53 %
Likelihood ratio = 43.7
Post test prob. of disease = 93.42 %
Pre test probability(%)
Likelihood ratio
Post test probability(%)
Pre test prob. of disease = 12.29 %
Likelihood ratio = 38.79
Post test prob. of disease = 84.46 %
Pre test probability(%)
Likelihood ratio
Post test probability(%)
Pre test prob. of disease = 10.41 %
Likelihood ratio = 10.65
Post test prob. of disease = 55.3 %

(j)

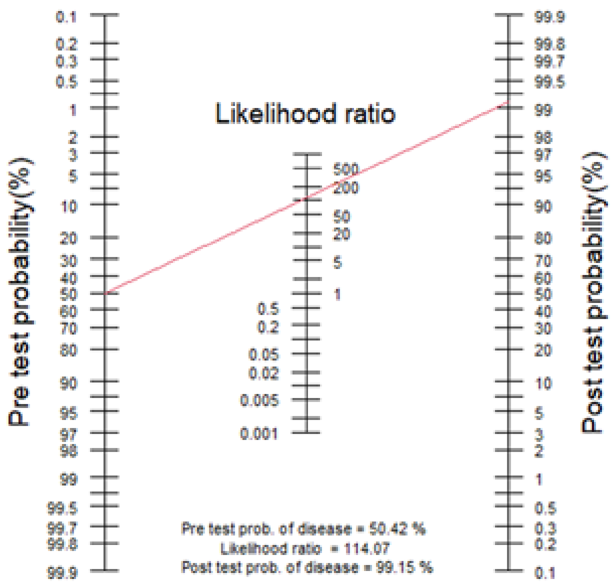

Supplementary Figure 4. Fagan's nomograms for: (a) Cen 2021, (b) Demir 2021, (c) Du 2021, (d) Li 2021b, (e) Li 2022, (f) Liu 2010, (g) Lu 2021a, (h) Lu 2021b, (i) Tan (2021) and (j) Tang 2022

References

Foo L, Ang M, Wong C et al. Is artificial intelligence a solution to the myopia pandemic?. *British Journal of Ophthalmology* 2021;105:741-744. doi:10.1136/bjophthalmol-2021-319129

Lin H, Long E, Ding X et al. Prediction of myopia development among Chinese school-aged children using refraction data from electronic medical records: A retrospective, multicentre machine learning study. *PLOS Medicine* 2018;15:e1002674. doi:10.1371/journal.pmed.1002674

Sun D, Du Y, Chen Q et al. Imaging Features by Machine Learning for Quantification of Optic Disc Changes and Impact on Choroidal Thickness in Young Myopic Patients. *Frontiers in Medicine* 2021;8. doi:10.3389/fmed.2021.657566

Yang X, Chen G, Qian Y et al. Prediction of Myopia in Adolescents through Machine Learning Methods. *International Journal of Environmental Research and Public Health* 2020;17:463. doi:10.3390/ijerph17020463

Shi C, Liu L, Wang Y et al. Research on screening system of myopic maculopathy based on deep convolution neural network. Chin J Exp Ophthalmol, 2021, 39(7): 602-608. doi: 10.3760/cma.j.cn115989-20191115-00495
